# Serum PCSK9 Correlates with PTX3 and Apolipoproteins B, A1, and C3 Concentrations in Patients with Type 2 Diabetes

**DOI:** 10.1155/2021/7956161

**Published:** 2021-12-21

**Authors:** Małgorzata Waluś-Miarka, Maria Kapusta, Przemysław Miarka, Ewa Kawalec, Barbara Idzior-Waluś

**Affiliations:** ^1^Department of Metabolic Diseases, Jagiellonian University Medical College, Jakubowskiego 2, 30-688 Krakow, Poland; ^2^University Hospital, Krakow, Poland; ^3^Department of Biochemistry, Jagiellonian University, Medical College, Poland; ^4^Department of Nephrology, Jagiellonian University Medical College, Poland

## Abstract

Proprotein convertase subtilisin/kexin type 9 (PCSK9) is involved in the regulation of LDL metabolism. There is evidence that circulating PCSK9 is a cardiovascular risk factor. In this study, we determined factors associated with circulating PCSK9 in a group of patients with type 2 diabetes mellitus (DM2). Material included 116 consecutive patients with DM2 from outpatient diabetes clinic. Circulating PCSK9, PTX3, apolipoprotein (apo) B100, apo B48, and apo C3 levels were determined by ELISA, apo A1 by immunoturbidimetry. The mean (sd) age of patients was 59.1 (11.1) years, the mean (sd) values of serum PCSK9 were 255.4 (106.97) ng/ml. Circulating PCSK9 correlated negatively with age (*r* = −0.21, *p* < 0.05) and HbA1c (*r* = −0.21, *p* < 0.05) and positively with BMI (*r* = 0.21, *p* < 0.05), total cholesterol (*r* = 0.59), LDL-cholesterol (*r* = 0.50), triglyceride (*r* = 0.35), apo B100 (*r* = 0.43), apo A1 (*r* = 0.43) (*p* < 0.001 for all), apo C3 (*r* = 0.29, *p* < 0.01), and apo B48 (*r* = 0.25, *p* < 0.01) concentration and FLI (*r* = 0.26, *p* < 0.01). Strong correlation between PTX3 and PCSK9 levels was observed (*r* = 0.47, *p* < 0.001). Multiple stepwise backward regression analysis with PCSK9 as dependent variable revealed that PTX3, apo B100, apo A1, apo B48, and BMI were significantly positive and the presence of NAFLD and HbA1c negatively associated with PCSK9 concentrations. These variables together explain 57% of PCSK9 variability; the strongest relationship was observed between PCSK9 and PTX3 and apo B100. Our results indicate that circulating PCSK9 is significantly associated with inflammation marker PTX3 as well as atherogenic lipids and apolipoproteins C3, B100, and B48, which might be of value in understanding interactions between development of atherosclerosis and inflammatory state in DM2 patients.

## 1. Introduction

Proprotein convertase subtilisin/kexin type 9 (PCSK9) has been gaining major attention after the emergence of data on the role of this protein in lipid homeostasis through regulation of low-density lipoprotein receptors and in atherosclerosis process. Increased PCSK9 is associated with decreased clearance of LDL-C and consequently with increased LDL-C serum concentration [1, 2]. There is gathering evidence that PCSK9 is the cardiovascular risk factor: the higher the PCSK9 levels, the higher the risk of atherosclerosis [3]. Moreover, data of Cheng et al. indicated that serum PCSK9 levels were associated with necrotic core tissue in coronary plaques, examined by IVUS imaging, independently of serum LDL-C concentrations [4]. However, there is still a controversy concerning the role of PCSK9 in predicting cardiovascular risk [5, 6]. Inflammation, which is involved in atherosclerotic processes, was found to stimulate the expression of PCSK9 in various cells. Furthermore, PCSK9 was found to have major impact on inflammatory process and is thought to be a critical regulator of innate immunity [7–10]. Recent data indicate that NRLP3 inflammasome via IL-1beta plays an important role in determining PCSK9 secretion [11]. The importance of PCSK9 to mediate type 2 diabetes has been recently demonstrated [10, 12]. Some studies show that higher PCSK9 levels are associated with higher fasting blood glucose and plasma insulin and insulin resistance [2, 12–14]. The data by Peyot et al. demonstrated a rise in the risk of developing glucose intolerance; however, recent data showed the opposite [15]. There is evidence that circulating PCSK9 increases with hepatic fat accumulation and correlates with the severity of steatosis, independently of metabolic confounders and liver damage [16, 17]. The recent data demonstrated that PCSK9 regulates hepatic triglyceride content in a manner dependent on CD36, scavenger receptor of fatty acids. Moreover, PCSK9 can protect against hepatic steatosis in the presence of excess dietary fats [18]. Interestingly, the loss-of-function PCSK9^Q152H^ variant carriers, apart from marked hypocholesterolemia, exhibited normal liver function despite their lifelong endoplasmic reticulum PCSK9 retention. Hepatoprotective effects of this variant could be explained by its cochaperone function [19].

Increasing number of data indicate that NAFLD is associated with cardiovascular risk [20, 21]. In young adult Korean persons, NAFLD was an independent predictor of MI and stroke [20]. According to recent meta-analysis, NAFLD is associated with increased long-term risk of cardiovascular events and might be an independent risk factor [21].

The results of clinical, experimental, and genetic data indicate that PCSK9 might be influenced by several metabolic and lipid risk factors, including atherogenic lipoproteins, obesity, and insulin resistance [12, 14].

In this study, we aimed to determine factors associated with circulating PCSK9 in a group of patients with DM2.

## 2. Material and Methods

In this observational, retrospective cross-sectional study, we enrolled consecutive patients with DM2, treated in outpatient diabetes clinic. Material included 116 consecutive patients, referred to diabetes outpatient clinic because of poor glycemic control. Diagnosis of diabetes was made based on EASD criteria. Patients with acute or chronic infections or devastating diseases were excluded. In each patient, a standardized questionnaire, including data on past history, treatment, presence of diabetes complication, diabetes duration, family history, and habits, was evaluated. In all patients, physical examination and anthropometric measurements were performed, using standardized methods.

Serum fasting lipid concentrations were determined by enzymatic methods (Cobas 8000 analyzer Roche Diagnostics, Mannheim, Germany) and HbA1c by HPLC (Bio-Rad Laboratories, Inc., Hercules, CA, USA). Human proprotein convertase subtilisin/kexin type 9 (PCSK9) and soluble low-density lipoprotein receptor (sLDLr) were determined by quantitative sandwich ELISA using a commercial reagent kit following the manufacturer's instruction (Human PCSK9 ELISA kit SEE189Hu, C*LOUD*-*CLONE CORP*., Houston, USA; Human LDLr ELISA kit SK00418-01, *AVISCERA BIOSCIENCE* Inc., Santa Clara, CA, USA) and PTX3 by sandwich ELISA using a commercial reagent kit following the manufacturer's instruction (#SEK411Hu: *Cloud-Clone Corp*., Houston, USA).

apo A1 was determined by immunoturbidimetry (APTEC Diagnostics nv, Belgium) and apo C3, apo B100, and apo B48 by sandwich ELISA kits (Human apo C3: # KA0465 Abnova Co., Taipei City, Taiwan, Human apo B100: SEA603Hu *Cloud-Clone Corp*., Houston, USA, and Human apo B48: #AKHB48 Shibayagi Co., Shibukawa, Gunma, Japan) according to the manufacturer's instructions. Fatty liver index (FLI) was calculated according to a formula using BMI, triglyceride concentration, GGTP, and waist circumference [22]. NAFLD was diagnosed by ultrasonography [23].

Statistical analysis included calculations of means (sd) or medians (interquartile range IR), when a variable was not normally distributed, and calculations of Spearman's (for variables not normally distributed) or Pearson's correlation coefficients. Student's *t*-test and Mann-Whitney *U* test were used to determine the differences between means (medians) of studied variables between patients with and without NAFLD and treated or not treated with statins. For variables, normally distributed analysis of variance ANOVA and Tukey's test were used to determine the differences between means in PCSK9 tertiles. For other variables, the Kruskal-Wallis test and test for multiple comparisons were used for comparison of distribution between PCSK9 tertiles. Regression analysis with PCSK9 as dependent variable, adjusted for age and sex, has been calculated in the whole examined group. The independent variables in regression analysis were body mass index (BMI), waist/hip ratio (WHR), systolic (SBP) and diastolic (DBP) blood pressure, serum concentration of total cholesterol (TC), triglycerides (TG), HDL-C, LDL-C, apolipoprotein B100, A1, C3, B48, sLDLr, glucose, creatinine, HbA1c, activity of alanine and asparagine aminotransferases (AST, ALT), and gamma-glutamyltranspeptidase (GGTP) for each variable separately. Multiple stepwise backward regression analysis was performed to determine the associations between examined variables and PCSK9, calculated in the whole examined group and in the subgroups divided according to the presence of NAFLD and statin treatment.

Written informed consent was obtained from each patient included in the study. The study protocol conforms to the local ethical guidelines and has been approved by the Jagiellonian University Collegium Medicum Ethics Committee on research on humans.

## 3. Results

We examined 116 patients with DM2, among them 57.8% were men. Mean (sd) age of patients was 59.1 (11.07) years, median (IR) duration of diabetes was 9 [10] years, mean (sd) values of HbA1c were 8.61 (2.32)%, and BMI was 32.74 (5.79) kg/m^2^. Mean (sd) values of serum LDL-cholesterol were 2.60 (1.16) mmol/l and of HDL-cholesterol 1.14 (0.30) mmol/l. Median (IR) of triglyceride concentration was 1.8(1.0) mmol/l. The more detailed biochemical characteristics of the patients were presented earlier [23].

100 patients (86.2%) had arterial hypertension, 79 (68.1%) hyperlipidemia, 79 (68.1%) NAFLD, and 43 (37.3%) coronary artery disease (CAD). 67 patients (59.8%) were treated with statins. Low physical activity was reported by 66 (56.9%) of patients, and 18 patients (15.5%) reported active smoking habit.

Table 1 presents mean (sd) values of PCSK9 serum concentrations according to sex, presence of NAFLD, presence of CAD, physical activity level, statin therapy, and smoking habit in examined patients. The mean (sd) value of serum PCSK9 concentration was 255.4 (106.97) ng/ml and was higher in women than in men (280.9 (118.87) vs. 236.8 (93.94)) ng/ml (*p* < 0.05). The mean PCSK9 concentrations did not differ between patients with and without NAFLD, smokers vs. nonsmokers, on statin therapy vs. nonstatin takers, nor with or without CAD.

Table 2 presents values of examined variables according to tertiles of PCSK9 serum concentrations. Total cholesterol increased from the lowest to the highest PCSK9 concentration tertiles (*p* < 0.001), while concentrations of LDL-C, apo B100, apo B48, apo C3, and PTX3 increased between tertiles 1-3 and 2-3, and apo A1 between the lowest and 2^nd^ and 3^rd^ tertile. Triglyceride and apo C3 concentrations, as well as FLI values, were highest in the third PCSK9 tertile. sLDLr concentration decreased from the lowest to the highest PCSK9 concentration tertiles (*p* < 0.001).

In the whole group of patients, circulating PCSK9 correlated negatively with age (*r* = −0.21, *p* < 0.05) and HbA1c (*r* = −0.21, *p* < 0.05) and positively with BMI (*r* = 0.21, *p* < 0.05), total cholesterol (TC) (*r* = 0.59, *p* < 0.001), LDL-cholesterol (*r* = 0.50, *p* < 0.001), and triglyceride (TG) (*r* = 0.35, *p* < 0.001). Negative correlation of PCSK9 with sLDLr concentration (*r* = −0.80, *p* < 0.001) was noted. We also found significant correlation between PCSK9 and apolipoproteins: apo B100 (*r* = 0.43, *p* < 0.001), apo B48 (*r* = 0.25, *p* < 0.01), apo A1 (*r* = 0.43, *p* < 0.001), and apo C3 (*r* = 0.29, *p* < 0.01) concentrations. Interestingly, strong positive correlation between PTX3 and PCSK9 was observed (*r* = 0.47, *p* < 0.001). We did not find any associations between PCSK9 and CK-18 fragments, nor liver enzymes, glucose, creatinine, and WHR; however, positive correlation between FLI, marker of liver steatosis, and PCSK9 was observed (*r* = 0.26, *p* < 0.01) (Table 3).

Correlation analysis revealed that in both groups of patients, either treated or not treated with statin, significant positive correlation between PCSK9 and TC, LDL-C, TG, PTX3, and apo B100 and negative with sLDLr concentrations was found. In the group of statin-treated patients, PCSK9 concentration was associated positively with apo A1 (*r* = 0.53, *p* < 0.001) and apo B48 (*r* = 0.24, *p* < 0.05) and negatively with serum creatinine (*r* = −0.32, *p* < 0.05). In patients not taking statins, negative correlation between HbA1c and PCSK9 was found (*r* = −0.45, *p* < 0.05) (Table 3).

In the NAFLD as well as non-NAFLD, patient associations between PCSK9 and total cholesterol, triglycerides, cholesterol LDL-C, sLDLr, apo B100, apo A1, PTX3, and FLI were found. Significant association between PCSK9 and apo C3 (*r* = 0.44, *p* < 0.001) and apo B48 (*r* = 0.23, *p* < 0.05) was observed only in NAFLD patients and between PCSK9 and BMI (*r* = 0.39, *p* < 0.05) was present only in a group without NAFLD (Table 3).

Regression and partial correlation analysis between PCSK9 and examined variables, after adjustment for age and sex, revealed that total and LDL-cholesterol, triglycerides, PTX3, apo B100, apo B48, apo C3, apo A1, and DBP were significantly positively associated with PCSK9 serum concentration (Table 4). sLDLr concentrations were negatively correlated with circulating PCSK9, while liver enzymes, glucose, BMI and WHR, CK-18, and FLI were not associated with PCSK9.

In the whole group of patients, multiple stepwise backward regression analysis with PCSK9 as dependent variable and age, apo B100, apo B48, apo A1, apo C3, PTX3, BMI, HbA1c, NAFLD, and statin treatment as independent variables revealed that PTX3, apo B100, apo A1, apo B48, and BMI were positively and presence of NAFLD and HbA1c negatively associated with PCSK9 concentrations (Table 5). These variables together explain 57% of PCSK9 variability; the strongest relationship was observed between PTX3 and PCSK9 (standardized regression coefficient was equal to 0.35) and between apo B100 and PCSK9 (standardized regression coefficient was equal to 0.34).

The multiple stepwise backward regression analysis was performed also in groups of patients treated and not treated with statins. In the group of patients treated with statins, the crude model consisted of PCSK9 as dependent variable and age, apo C3, apo B48, apo B100, apo A1, and PTX3 as independent variables. It was shown that PTX3, apo A1, and apo B100 were significantly associated with PCSK9 (Table 6). These variables together explain 51% of PCSK9 variability; the strongest relationship was observed between PTX3 and PCSK9 (standardized regression coefficient was equal to 0.44) and between apo A1 and PCSK9 (standardized regression coefficient was equal to 0.41). In the group of patients not treated with statins, the crude model consisted of PCSK9 as a dependent variable and apo C3, PTX3, apo A1, and apo B100 as independent variables. It turned out that PTX3 and apo C3 were significantly associated with PCSK9. These two variables explain the 30% variability of PCSK9, and PTX3 was the strongest predictor (standardized regression coefficient equal to 0.41) (Table 6).

In the group of patients with NAFLD, multiple stepwise backward regression analysis with PCSK9 as dependent variable and age, apo C3, PTX3, apo B48, apo B100, and apo A1 as independent variables revealed that apo A1, apo C3, apo B100, and PTX3 were significantly associated with PCSK9 (Table 6). These variables together explain 47% of PCSK9 variability; the strongest relationship was observed between apo A1 and PCSK9 (standardized regression coefficient was equal to 0.33). In the group of patients without NAFLD, multiple stepwise backward regression analysis with PCSK9 as dependent variable and PTX3, apo B48, apo B100, BMI, and apo A1 as independent variables revealed that PTX3, apo B48, and apo A1 were significantly associated with PCSK9. These variables together explain 49% of PCSK9 variability; the strongest relationship was observed between PTX3 and PCSK9 (standardized regression coefficient was equal to 0.54) (Table 6).

## 4. Discussion

Increasing number of data indicate that high levels of PCSK9 are associated with proatherosclerotic processes; therefore, the factors associated with PCSK9 concentrations are the subjects of intensive research [2–4]. In our cohort of type 2 diabetes patients, we extended previously described associates of serum PCSK9 concentrations with lipids on apolipoproteins: apo B100, apo A1, apo C3, and apo B48, as well as on inflammation marker PTX3. All these parameters were highest in the highest tertile of PCSK9 concentrations and significantly positively correlated with circulating PCSK9.

We confirmed observed by others positive associations between PCSK9 and total cholesterol, LDL-cholesterol, and triglyceride [14, 24–26], although Mayne et al. observed correlation with cholesterol in men only [27], while Lambert et al. and Caselli et al. did not observe associations between PCSK9 and triglyceride [24, 28].

Correlations between apolipoprotein B100, the main apolipoprotein of LDL, and other liver-derived proatherogenic lipoproteins were also observed in patients with stable angina, in patients with high cardiovascular risk and in population of young patients [26, 28, 29]. Interestingly, PCSK9 is believed to play a role in the metabolism of apo B containing lipoproteins from the liver and modulation of lipogenesis [17, 30]. Experimental studies indicate that PCSK9 deficiency reduces apolipoprotein B secretion and modulates atherogenic lipoproteins, independently of LDL receptor [30].

Apolipoproteins B48 and C3, markers of triglyceride-rich lipoproteins, are involved in atherosclerotic/inflammatory processes and increase vascular complications [31–34]. apo C3 recently has emerged as novel therapeutic target for correction of dyslipidemia and decrease of cardiovascular risk [32, 33]. Apo B48, a marker of intestinally derived lipoproteins, correlated significantly positively with circulating PCSK9 in fasting state in our DM2 patients. On the other hand, in the study of Chan et al., plasma PCSK9 concentrations were associated with the postprandial apo B48 concentrations in obese persons, in line with our data [31]. In this study, the correlation between apo C3 and PCSK9 concentrations was observed in the whole group of DM2 patients as well as in patients with NAFLD only, which might support the role of PCSK9 and apo C3 in the pathogenesis of cardiovascular disease in NAFLD diabetic patients.

In type 2 DM patients, we observed significant positive association between PCSK9 and apo A1, the main apolipoprotein of HDL lipoproteins. Positive correlations between PCSK9 and HDL-C and apo A1 were observed in adult populations; however, we did not observe in opposite to others any associations between HDL-C and PCSK9 [26, 28, 29]. Lower HDL-C levels, as well as lower apo A1 serum concentration, were observed in the lowest quartile of PCSK9 serum concentrations in line with our data on apo A1 in a cohort of patients with CAD enrolled in the EVINCI study, which included 30% of patients with DM and in young persons with the presence of metabolic markers [28, 29]. The association between PCSK9 and apo A1 was present in the whole group of patients in patients with and without NAFLD. However, when we looked at a group of patients treated and not treated with statins, the PCSK9 and apoA1 association was present only in the subgroup of statin-treated patients, while statin treatment had no effect on the tight correlation of PCSK9 levels with apo B concentration. In the statin-treated group, apo A1, apart from PTX3 and apo B100, was strongly associated with PCSK9, while in patients not using statins, the association of apo A1 with PCSK9 was abolished.

In this study, PCSK9 correlated significantly with PTX3 in the total group as well as in all subgroups of patients. Recent observations suggest that PCSK9 is involved in inflammation processes with implications in atherosclerosis and its major consequence—myocardial ischemia [7, 8, 35, 36]. Ding et al. reported elevated serum PCSK9 levels in patients with myocardial ischemia and significant correlation between serum levels of PCSK9 and proinflammatory cytokines—IL-6, IL-1b, TNFa, and MCSF as well as hsCRP [9]. Moreover, PCSK9 positively correlated with C-reactive protein [14, 37] and white blood cell number indicating a role in the immune response [37]. In this study, PTX3, an essential component of innate immunity, and a novel cardiovascular risk factor, also in patients with type 2 diabetes [38–41] was the strongest predictor of PCSK9 levels in multiple stepwise regression analysis.

Statins have been found to upregulate PCSK9 expression and thereby reducing their own lipid-lowering effects [42]. In our study, patients receiving statins had as expected lower LDL-C than patients not treated with statins but did not differ regarding PCSK9 concentration. Interestingly, in patients treated with statins, PTX3, apo A1, and apo B100 explained 51% of PCSK9 variability. In this group, the strongest relationship was observed between PCSK9 and PTX3.

In our patients, sLDLr concentration correlated strongly negatively with PCSK9 levels, in opposite to the observation of Mayne et al. who observed in a white Canadian cohort significant positive association between sLDLr and PCSK9 [43]. On the other hand, Girona et al. did not observe any associations between sLDLr and PCSK9 in children [44].

There are some data that PCSK9 is involved in NAFLD development [16, 17]. We observed negative association in multiple regression analysis between the presence of NAFLD and PCSK9 concentration in line with data of Wargny et al. who did not find associations between circulating PCSK9 and severity of hepatic steatosis [45]. In our study, PCSK9 concentrations did not correlate with liver enzymes, similarly like in Wargny et al.'s study, nor with CK-18 fragments, a marker of NAFLD [45]. Moreover, animal data as well as recently published studies with loss-of-function PCSK9Q152H variant indicate a protective role of PCSK9 in NAFLD development [17, 18, 46]. However, we observed association of PCSK9 with the FLI index, a marker of NAFLD, which disappeared after adjustment by age and sex. In opposite to our data, Paquette et al. found associations between PCSK9 and liver enzymes [47].

The recent literature underlines that PCSK9 plays an important role in glucose homeostasis, influencing the survival and normal function of pancreatic islet beta cells [2]. Our observations on negative association between HbA1c and PCSK9 are opposite to data from a cohort of the Dallas Heart Study, in which a significant correlation among PCSK9 and fasting serum glucose, insulin, and HOMA-IR has been found [14].

PCSK9 levels were positively associated with BMI in the whole group of our patients, similarly like in other studies [14], but not all authors observed such associations [17, 24, 29]. Observations that calorically restricted diets were associated with decreasing PCSK9 concentrations that are in line with our findings [47, 48].

In the literature data, serum PCSK9 concentration correlated positively with age in patients suspected for NAFLD and in adolescents [17, 29]. In our patients, negative correlation between age and PCSK9 concentration in the whole group of DM2 patients and in subgroup with NAFLD was observed. In this study, PCSK9 serum levels were higher in women than in men, in line with Lakoski et al. in a multinational cohort [14].

The limitation of this study is relatively small number of patients. We did not perform other inflammation markers; we plan to perform them in future studies. The strength of the study is a representative group of DM2 patients, who are at high risk of vascular disease and NAFLD and use of apolipoprotein, which are according to some data better than lipoprotein risk markers associated with atherosclerosis.

The results of our study indicate that there are strong positive associations between circulating PCSK9 and atherogenic apolipoproteins B100, C3, and B48, as well as unexpectedly antiatherogenic apo A1 serum concentrations. These data might be of value in understanding the pathophysiology of lipid metabolism according to statin treatment and presence of NAFLD in patients with diabetes and could be of value in modulation of CVD risk in DM2 patients. Association of PCSK9 with PTX3, an inflammatory marker, might suggest the important role of PCSK9 in inflammatory atherosclerotic processes.

## Figures and Tables

**Table 1:** Mean values of PCSK9 serum concentrations (ng/ml) in patients with type 2 diabetes mellitus according to sex, CAD, smoking status, statin therapy, and physical activity.

Parameter		*n*	*x*	sd	*p*
Sex	Male	67	236.8	93.94	0.0274
	Female	49	280.9	118.87	
CAD	Present	43	245.9	112.75	0.4652
	Absent	73	261.0	103.80	
NAFLD	Present	79	244.0	93.48	0.1362
	Absent	37	279.9	129.29	
Smoker	Regular	18	224.7	109.88	0.1854
	Former or never	98	261.1	106.02	
Statin therapy	Yes	67	248.6	99.90	0.2488
	No	45	272.1	112.47	
Physical activity	Low	66	243.8	100.00	0.1782
	Average or high	50	270.8	113.60	

**Table 2:** Characteristics of examined variables according to PCSK9 tertile concentrations.

Parameters	Tertiles PCSK9									*p*
	1 (*N* = 38)			2 (*N* = 39)			3 (*N* = 39)			
	PCSK9 ≤ 200 ng/ml			200 ng/ml < PCSK9 ≤ 280 ng/ml			PCSK9 > 280 ng/ml			
	*n*	*x*/Me	sd/IR	*n*	*x*/Me	sd/IR	*n*	*x*/Me	sd/IR	
Age (yrs)	38	62.0	10.18	39	59.3	10.03	39	56.1	12.30	0.0619
Diabetes duration (yrs)^∗^	38	11.7	9.15	39	7.3	6.13	37	8.9	6.29	0.0308
HbA1c (%)	31	9.01	2.50	29	8.69	2.40	27	8.07	1.99	0.3044
Waist circumference (cm)	38	109.5	11.71	38	106.2	12.86	37	111.7	14.39	0.1802
BMI (kg/m^2^)	38	31.6	4.44	39	32.3	5.64	39	34.3	6.80	0.0968
SBP (mmHg)	38	129.0	15.95	39	130.8	18.91	38	131.4	19.63	0.8379
DBP (mmHg)	38	79.0	10.81	39	81.5	8.33	38	81.8	14.24	0.4896
TC (mmol/l)^&^	35	3.80	1.20	34	4.60	1.20	37	5.50	1.50	<0.001
TG (mmol/l)^∗^	35	1.50	0.84	34	1.62	1.10	37	2.06	1.30	0.0062
HDL-cholesterol (mmol/l)	35	1.11	0.292	34	1.14	0.374	37	1.16	0.236	0.8390
LDL-cholesterol (mmol/l)	35	2.03	0.725	32	2.45	0.908	31	3.42	1.349	<0.001
Glucose (mmol/l)	32	7.77	2.880	27	7.03	2.678	32	7.94	2.059	0.3595
Creatinine (mmol/l)^∗^	38	76.50	22.00	35	78.00	25.00	35	75.00	25.00	0.4548
Uric acid (*μ*mol/l)	35	322	86.07	33	359	102.19	35	360	94.09	0.1642
PTX3 (ng/ml)^∗^	38	3.23	1.73	39	4.02	2.39	39	5.25	2.25	<0.001
apo B48 (ng/ml)^∗^	38	3417.5	2358.0	39	3695.0	2897.0	38	5241.5	3619.0	0.0167
apo B100 (mg/dl)	38	76.9	18.62	39	85.2	24.05	39	104.1	26.53	<0.001
apo A1 (mg/dl)	38	126.9	29.67	39	143.0	20.46	39	154.4	29.03	<0.001
apo C3 (*μ*g/dl)^∗^	38	209.0	135.2	39	209.3	136.1	38	283.9	146.4	0.0029
sLDLr (ng/ml)	38	165.3	21.10	39	123.6	17.46	39	100.9	19.63	<0.001
FLI^∗^	34	82.69	23.30	33	85.33	31.54	33	92.54	19.92	0.0472
AST (U/L)^∗^	38	25.00	15.00	35	24.00	16.00	36	25.00	13.50	0.9424
ALT (U/L) ^∗^	38	28.00	29.00	35	26.00	22.00	37	31.00	25.00	0.9889
GGTP (U/L) ^∗^	36	29.00	20.50	35	28.00	27.00	35	30.00	42.00	0.5769

^∗^Due to the lack of normality of the distribution, the median (Me) and the interquartile range (IR) were given, and the Kruskal-Wallis test was used to assess the differences in distributions between PCSK9 tertiles; in the remaining cases, the mean (*x*) and standard deviation (sd) were given, and the ANOVA analysis of variance was used to evaluate the differences in the mean values between PCSK9 tertiles. ^&^Due to the lack of homogeneity of the TC variance between PCSK9 tertiles, Me and IR were given for the description of TC, and the Kruskal-Wallis test was used to evaluate the differences between the distributions.

**Table 3:** Pearson's and Spearman's^&^ correlation coefficients between PCSK9 and chosen variables in patients with type 2 diabetes in the studied group and according to statin treatment and presence of NAFLD.

Parameter	Total	Statin therapy			Nonalcoholic fatty liver disease
	*N* = 116	Yes (*N* = 67)	No (*N* = 45)	Present (*N* = 79)	Absent (*N* = 37)
Age (yrs)	-0.21^∗^	-0.15	-0.17	-0.24^∗^	-0.29
Diabetes duration (yrs)	-0.11^&^	0.01	-0.15^&^	-0.16^&^	-0.11^&^
HbA1c (%)	-0.21^∗^	-0.02	-0.45^∗^	-0.23	-0.23
Waist circumference (cm)	0.10	-0.04	0.15	-0.01	0.32
WHR	-0.10	-0.22	0.09	-0.06	-0.11
BMI (kg/m^2^)	0.21^∗^	0.06	0.22	0.12	0.39^∗^
SBP (mmHg)	0.04	0.01	0.13	0.02	-0.03
DBP (mmHg)	0.13	-0.01	0.23	0.21	0.01
TC (mmol/l)	0.59^∗∗∗^	0.51^∗∗∗^	0.64^∗∗∗^	0.57^∗∗∗^	0.60^∗∗∗^
TG (mmol/l)	0.35^&^^∗∗∗^	0.26^&^^∗^	0.43^&^^∗∗^	0.39^&^^∗∗∗^	0.41^&^^∗^
HDL-C (mmol/l)	0.04	0.24	-0.22	-0.04	0.01
LDL-C (mmol/l)	0.50^∗∗∗^	0.45^∗∗∗^	0.56^∗∗∗^	0.46^∗∗∗^	0.51^∗∗^
Glucose (mmol/l)	0.07^&^	0.26^&^	-0.24	0.03	0.08^&^
Creatinine (*μ*mol/l)	-0.10^&^	-0.32^&^^∗^	0.22	-0.08^&^	-0.16
Uric acid (*μ*mol/l)	0.11	0.07	0.11	0.14	0.13
Bilirubin (*μ*g/dl)	-0.12	-0.10	-0.13	-0.02	-0.23
CK-18 (U/l)	0.06^&^	0.05^&^	0.10^&^	0.09^&^	0.09^&^
PTX3 (ng/ml)	0.47^&^^∗∗∗^	0.42^&^^∗∗∗^	0.52^&^^∗∗∗^	0.41^∗∗∗^	0.48^&^^∗∗^
apo B48 (ng/ml)	0.25^&^^∗∗^	0.24^&^^∗^	0.13^&^	0.23^&^^∗^	0.26^&^
apo B100 (mg/dl)	0.43^∗∗∗^	0.39^∗∗^	0.44^∗∗^	0.49^∗∗∗^	0.34^∗^
apo A1 (mg/dl)	0.43^∗∗∗^	0.53^∗∗∗^	0.23	0.46^∗∗∗^	0.38^∗^
apo C3 (*μ*g/dl)	0.29^&^^∗∗^	0.20	0.24^&^	0.44^&^^∗∗∗^	0.06
sLDLr (ng/ml)	-0.80^∗∗∗^	-0.77^∗∗∗^	-0.80^∗∗∗^	-0.84^∗∗∗^	-0.75^∗∗^
FLI	0.26^&^^∗∗^	0.07^&^	0.36^&^^∗^	0.28^&^^∗^	0.38^&^^∗^
AST (U/l)	-0.05^&^	-0.14^&^	0.11^&^	-0.01^&^	-0.07^&^
ALT (U/l)	-0.03^&^	-0.14^&^	0.08^&^	-0.03^&^	0.01^&^
GGTP (U/l)	-0.01^&^	-0.09^&^	0.08^&^	-0.08^&^	-0.05^&^

^∗^ *p* < 0.05, ^∗∗^*p* < 0.01, and ^∗∗∗^*p* < 0.001; ^&^Spearman correlation coefficient.

**Table 4:** Regression and partial correlation coefficients between examined variables and PCSK9 in patients with type 2 diabetes mellitus, after adjustment for age and sex.

Parameter	*N* = 116				
	*n*	*b*	*r*	*R* ^2^	*p*
Diabetes duration (yrs)	114	-1.019	-0.07	0.13	0.4594
HbA1c (%)	87	-9.608	-0.21	0.03	0.0533
Waist circumference (cm)	113	0.923	0.12	0.04	0.2125
WHR	112	94.642	0.06	0.37	0.5557
BMI (kg/m^2^)	116	2.705	0.15	0.04	0.1061
SBP (mmHg)	115	1.028	0.17	0.15	0.0674
DBP (mmHg)	115	1.781	0.20	0.03	0.0342
TC (mmol/l)	106	43.398	0.56	0.07	<0.001
TG (mmol/l)	106	35.796	0.43	0.05	<0.001
HDL-C (mmol/l)	106	14.826	0.04	0.06	0.6688
LDL-C (mmol/l)	98	38.147	0.45	0.08	<0.001
Glucose (mmol/l)	91	0.687	0.02	0.01	0.8747
Creatinine (mmol/l)	108	0.302	0.07	0.15	0.4685
Uric acid (*μ*mol/l)	103	0.173	0.16	0.05	0.1217
Bilirubin (*μ*g/dl)	95	-1.814	-0.08	0.06	0.4235
CK-18 (U/l)	116	0.011	0.06	0.10	0.5384
PTX3 (ng/ml)	116	16.033	0.46	0.05	<0.001
apo B48 (ng/ml)	115	0.008	0.31	0.02	0.0008
apo B100 (mg/dl)	116	1.681	0.43	0.01	<0.001
apo A1 (mg/dl)	116	1.623	0.46	0.00	<0.001
apo C3 (*μ*g/dl)	115	0.260	0.30	0.06	0.0013
sLDLr (ng/ml)	116	-2.478	-0.79	0.04	<0.001
FLI	100	0.751	0.16	0.03	0.1179
AST (U/l)	109	-0.153	-0.04	0.11	0.6539
ALT (U/l)	110	-0.202	-0.07	0.17	0.4601
GGTP (U/l)	106	-0.033	-0.01	0.10	0.9027

*b*: partial regression coefficient; *r*: partial correlation coefficient; *R*^2^: partial determination coefficient.

**Table 5:** Results of multiple backward stepwise regression analysis with PCSK9 as dependent variable in the whole group of patients.

Variables in the reduced model	*b* ^∗^	**b**	se	*R* ^2^	**p**
PTX3 (ng/ml)	0.35	12.55	2.860	0.13	<0.001
apo B100 (mg/dl)	0.34	1.43	0.350	0.20	0.0001
BMI (kg/m^2^)	0.26	5.79	1.777	0.16	0.0017
apo A1 (mg/dl)	0.25	1.01	0.319	0.16	0.0023
NAFLD	-0.19	-48.33	18.938	0.06	0.0128
apo B48 (ng/ml)	0.17	0.004	0.002	0.13	0.0375
HbA1c (%)	-0.17	-8.12	3.848	0.12	0.0384

*b*: partial regression coefficient; *b*^∗^: partial standardized regression coefficient; *r*: partial correlation coefficient; se: standard error for regression coefficient; *R*^2^: partial determination coefficient.

**Table 6:** Results of multiple backward stepwise regression analysis with PCSK9 as dependent variable in group of patients according to statin treatment and according to presence of NAFLD.

Variables in the reduced model	*b* ^∗^	**b**	se	*R* ^2^	**p**
*On statins*					
PTX3 (ng/ml)	0.44	13.75	2.774	0.03	<0.001
apo A1 (mg/dl)	0.41	1.54	0.340	0.09	<0.001
apo B100 (mg/dl)	0.21	1.01	0.442	0.09	0.0254
*No statins*					
PTX3 (ng/ml)	0.41	16.54	5.172	0.03	0.0026
apo C3 (*μ*g/ml)	0.34	0.28	0.107	0.03	0.0121
*NAFLD present*					
apo A1 (mg/dl)	0.33	1.06	0.285	0.09	0.0004
apo C3 (*μ*g/dl)	0.26	0.19	0.068	0.24	0.0065
apo B100 (mg/dl)	0.25	0.87	0.321	0.20	0.0082
PTX3 (ng/ml)	0.21	10.89	4.866	0.19	0.0282
*No NAFLD*					
PTX3 (ng/ml)	0.54	15.21	3.380	0.005	<0.001
apo B48 (ng/ml)	0.32	0.010	0.004	0.06	0.0136
apo A1 (mg/dl)	0.26	1.15	0.542	0.06	0.0408

*b*: partial regression coefficient; *b*^∗^: partial standardized regression coefficient; *r*: partial correlation coefficient; se: standard error for regression coefficient; *R*^2^: partial determination coefficient.

## Data Availability

The data that support the findings of this study are available on request from the corresponding author.
